# A Community-Up Approach to Improve Health and Wellbeing in a Dutch Neighbourhood. A Case Study

**DOI:** 10.5334/ijic.9811

**Published:** 2026-05-22

**Authors:** Leendert J. Guijt, Petra C. Siemonsma, Arlette E. Hesselink, Suzan Van Der Pas

**Affiliations:** 1 Research Group Physical Activity, Self-Management and Behaviour Change, University of Applied Sciences Leiden, Leiden, NL; 2 Research Group Social Innovation, University of Applied Sciences Leiden, Leiden, NL; 3 Department of Public Health and Primary Care/Health Campus the Hague, Leiden University Medical Centre, the Hague, NL

**Keywords:** integrated people-centred health care services, integrated care, community-up, community engagement, neighbourhood

## Abstract

**Introduction:**

This case study describes a novel community-up approach designed to gain insight into the needs and aspirations of both residents and professionals in a Dutch neighbourhood, aiming to enhance health and wellbeing. The Integrated People-Centred Health Care Services (IPCHS) framework served as the theoretical lens for evaluating the findings.

**Method:**

A total of 150 residents and 50 professionals from sectors, including health care, social services, and municipal governance participated. Using participatory action research (PAR), researchers explored local needs and aspirations through a variety of methods, including participatory observations, (in-depth) interviews, transect walks, two world café meetings, and desk research.

**Results:**

Needs, aspirations, and opportunities were synthesized into an *Atlas of the Neighbourhood* and a *Treasure Map*. Beyond these outcomes, several valuable unintended effects emerged, including stronger relationships, increased community engagement, and improvements aligned with the IPCHS framework.

**Discussion:**

This study illustrates the potential of a novel community-up, PAR-based approach, integrating diverse, generative methods to foster meaningful connections among residents and professionals. It also underscores the added value of applied research, particularly the role of University of Applied Sciences researchers as critical allies in engaging residents, local professionals, and stakeholders such as directors and policymakers.

## Introduction

High-income countries are increasingly confronted with health and social challenges, including ageing populations, with a rising prevalence of chronic diseases, widening health inequalities, and escalating healthcare costs [1,2,3]. Individuals and communities are increasingly encouraged to take greater ownership of their health and to actively participate in efforts to improve health outcomes for all [4,5]. To maintain universal health coverage, promote health equality, and ensure the sustainability of health systems, there is a shift away from a reactive illness-focused model at an individual level towards a more holistic and proactive approach that supports the health and wellbeing of entire communities [2,6,7,8,9]. Importantly, health and wellbeing are shaped by a range of social determinants beyond the healthcare system, such as access to healthy food, opportunities for physical activity, and enough financial resources [5,10,11].

Both the physical and social aspects of the neighbourhood – as the immediate environment of individuals – greatly impact residents’ health and behaviours [11,12,13,14,15]. Contributing physical aspects include the availability of sports facilities, health and wellbeing services, healthy food options, and walkability of the neighbourhood [12,13,14]. Contributing social aspects include the availability of networks and support, relationships, and a sense of belonging. Interventions aimed to improve health and wellbeing in the neighbourhood, if needed, should focus on the physical and social environment and unique health needs and aspirations of both residents and professionals [16].

### Theoretical background

To meet the various health and social challenges, the World Health Organization (WHO) introduced the Integrated, People-Centred Health Care Services (IPCHS) framework [1,17]. This framework is based on the core values and principles of primary health care: “a whole-of-society approach to health that aims equitably to maximize the level and distribution of health and wellbeing by focusing on people’s needs and preferences (both as individuals and communities) as early as possible along the continuum from health promotion and disease prevention to treatment, rehabilitation and palliative care, and as close as feasible to people’s everyday environment” [6,18].

The IPCHS framework supports a fundamental change in the way health services are funded, managed, and delivered – notably, a shift away from health systems designed around diseases and health institutions to health systems designed with and for people, respecting their needs and aspirations [1,2,17,19,20,21]. Given that health systems are highly context-specific, the framework does not suggest a universal model of people-centred, integrated health services. Instead, it outlines five interdependent strategies that should be pursued: (1) engaging and empowering individuals and communities; (2) enhancing governance and accountability; (3) reorienting the model of care; (4) facilitating service coordination within and across sectors; and (5) establishing a supportive environment and ensuring financial sustainability [1,2].

The implementation of each IPCHS strategy should be locally designed and developed, taking into account the specific context, policies, and the needs and aspirations of both residents and professionals [1,2,17]. To our knowledge, while a limited number of studies have explored approaches for identifying local needs and aspirations in collaboration with residents and professionals, none have described how to integrate these needs and aspirations into the IPCHS framework [20,22].

This case study describes a novel community-up approach to gain insight into the needs and aspirations of both residents and professionals in a Dutch urban neighbourhood, Leiden Zuidwest, and opportunities enhancing health and wellbeing. In this study, a community-up approach refers to an approach in which the needs, aspirations, and assets of residents and professionals are first collaboratively explored and then used as a guiding basis for strengthening health and wellbeing in the neighbourhood, rather than following predefined institutional agendas [23]. Participatory action research (PAR) was employed to support a community-up approach. PAR is a cyclical and practice-based research approach that combines participation with iterative processes of reflection and action aimed at practical change. It emphasises collaboration between residents and professionals through collective co-creation and co-learning, using methods that actively involve participants from the outset [24,25]. The IPCHS framework was applied retrospectively as a theoretical lens to evaluate and interpret the results. This study explores the value of the adopted approach, using the case as an illustrative example.

## Method

### Case description

The Netherlands places strong emphasis on delivering people-centred, integrated care that aligns with the needs and preferences of both residents and professionals at neighbourhood level [26,27,28,29]. In 2023, the Dutch government, municipalities and health insurers formalized this commitment in the Healthy and Active Living Agreement. This agreement outlines local and regional goals of prevention, health and (social) wellbeing [26] aiming to achieve a healthy generation by 2040, fostering resilient, healthy people who can grow up, live, and work in a supportive and health-promoting environment, underpinned by a robust social safety net. Municipalities are responsible for implementing local collective prevention efforts, promoting social cohesion, participation, social support, and a healthy living environment [30]. Health insurers are responsible for ensuring that individuals have access to necessary care, which is provided by healthcare organizations and professionals. At the same time, individuals are expected to take responsibility for their own health and lifestyle choices, and are encouraged to utilise their personal capabilities and social networks.

The city of Leiden in the Netherlands has a population of 130,067 in 2024 and is characterized by a high population density [31]. To improve the health of its residents, the municipality has established a local prevention agreement in collaboration with various city stakeholders [32]. This initiative aims to provide all residents with opportunities to live and work in optimal health, with a particular attention to districts with a relatively high prevalence of health and social issues.

Leiden Zuidwest is a post-war, densely populated neighbourhood with a population of over 20,000 [31]. It is characterized by a high degree of diversity in terms of cultural background, age, and income level [33,34]. Compared to other parts of the city, the neighbourhood faces relatively higher levels of health and social challenges, including a greater prevalence of chronic conditions, social vulnerability, and socioeconomic inequalities [32,33,34]. These interconnected challenges highlight the need for coordinated action at neighbourhood level.

Primary health care services are delivered through a neighbourhood partnership comprising all primary care providers – such as general practitioners, nurses, pharmacists, and allied health professionals, along with four welfare organisations. Additionally, other key stakeholders, including policymakers, local entrepreneurs, police officers, and housing corporations, are involved due to their direct or indirect influence on the health and wellbeing of residents. Strengthening health and wellbeing in Leiden Zuidwest therefore requires collaboration across health, social, and community sectors.

In response to these challenges, and in alignment with municipal and national policy ambitions, this study aimed to explore how health and wellbeing could be strengthened at neighbourhood level. While the overarching ambition—to improve health and wellbeing—was policy-aligned, the specific focus of the study was intentionally left open. Rather than predefining themes or target groups, residents and professionals were invited to articulate their own needs, aspirations, and perceived challenges, thereby shaping the direction and substance of the research from the pre-orientation phase onwards.

The University of Applied Sciences Leiden (UASL) and the municipality of Leiden share a common goal: to improve health and wellbeing at the neighbourhood level, guided by the needs and aspirations of local residents and professionals. At UASL, researchers conduct practice-oriented studies that have a direct impact on society and generate new scientific insights. In addition to their research, UASL researchers can act as independent allies to residents and professionals. This case study was conducted by a team of researchers from the UASL with competences in health and in social work. One team member also practices as a physiotherapist in an adjacent neighbourhood of Leiden. Moreover, physiotherapy students contributed actively to the project, working under the supervision of the research team.

### Description of the approach

The study was carried out between June 2023 and June 2024 and was structured into three distinct phases, based on the principles of PAR [25]; 1. pre-orientation, 2. orientation, and 3. planning (see Table 1). The study focused on the exploratory, orientation phases of PAR. The later action and evaluation phases of the full PAR process extend beyond the scope of the present case study. Each phase had its own specific purpose, methods, and participants, although with some overlap across the phases.

**Table 1:** The three phases of this PAR study.


PHASE	1. PRE-ORIENTATION	2. ORIENTATION	3. PLANNING

**Period**	June ’23 –Jan ‘24	Aug ’23 – May ‘24	Nov ’23 – May ‘24

**Purpose**	Exploring and understanding local ecosystem and collecting opinions and thoughts of residents and professionals in relation to health and wellbeing	Identifying needs, aspirations and exploring opportunities towards a health and wellbeing neighbourhood and applying public scrutiny	Exploring common ground and starting points of some opportunities to improve health and wellbeing in Leiden Zuidwest

**Methods**	Observational research, unstructured and structured interviews & desk research	Unstructured and structured in-depth interviews, transect walks and two Dream Laboratories	Visualize results for reflection and participants’ check

**Participants**	Residents (n = 150), health and social professionals and other local stakeholders such as municipal policymakers, police officers, housing corporations, and entrepreneurs) (n = 50)	Residents (n = 50), health and social professionals and other local stakeholders, such as municipal policymakers, police officers, housing corporations, and entrepreneurs (n = 30)	Residents (n = 50), health and social professionals and other local stakeholders, such as municipal policymakers, police officers, housing corporations, and entrepreneurs (n = 30)


In the **pre-orientation phase**, the local ecosystem was explored to gain familiarity with the neighbourhood, understand its physical layout, engage with residents and professionals, and collect their perspectives on health and wellbeing in the neighbourhood. A combination of observational research, interviews (both unstructured and structured), and desk research was employed. Observational research consisted of researchers walking and cycling through the neighbourhood at various times and days, taking pictures and making notes to document neighbourhood characteristics. Additionally, to get an idea of the community’s perceptions on health and wellbeing, researchers engaged in informal conversations and conducted structured and unstructured interviews with residents, local health and social care professionals, municipal policymakers, police officers, housing corporations, and entrepreneurs. Residents were approached by researchers and students at various times and locations, including in public spaces, streets, community centres, through professional contacts, and during local events and activities such as iftar dinners, community parties, creativity clubs, and sport gatherings. To ensure accessibility to residents, data was collected note-taking rather than audio or video recordings. Desk research involved collecting both quantitative and qualitative data on demographics, health status, lifestyle factors, and the availability of local primary health care services. Sources included policy documents and publicly accessible databases maintained by the municipality and local welfare organisations, containing neighbourhood statistics, demographic data, and reports on social and health-related initiatives. Local newsletters and community leaflets were also reviewed, such as publications produced by welfare organisations and volunteer groups. Additional sources included newspapers, community resources, site visits, and websites of relevant stakeholders, such as the municipality, health and wellbeing organisations, local entrepreneurs, police, and housing corporations.

The **orientation phase** pursued two goals: (1) to identify needs and aspirations of, and opportunities for, residents and professionals to improve health and wellbeing, and (2) to apply public scrutiny. Information was collected through both qualitative and generative research methods, like unstructured and structured in-depth interviews, transect walks and two world café meetings called Dream Laboratories (*DromenLab* in Dutch). Professionals were interviewed either individually or together with colleagues; residents shared their perceptions in a variety of local contexts. Transect walks provide a participatory method in which residents select routes through the neighbourhood, reflecting on local strengths, barriers, and areas for improvement while walking. The Dream Laboratories sessions brought together residents, health and wellbeing professionals, and other stakeholders—including municipal policymakers, police officers, housing corporations, and entrepreneurs—to apply public scrutiny and to gain deeper insights into the collected needs and aspirations. Each session began with an interactive presentation of the latest exploration findings. To delve deeper into the data and prioritise preliminary insights, participants were then divided across themed world café tables – a participatory dialogue method that facilitates small-group discussions around key themes, enabling participants to exchange ideas, reflect collectively, and co-create shared insights. Depending on each table’s focus and objectives, various generative methodologies were used, including dreaming (inviting participants to envision an ideal future situation and explore what their community could become, stimulating creativity, shared aspirations, and transformative thinking [23,35]), brainstorming, perspective shifting, and ‘what if’ scenarios. Data were collected through notes on post-its and a concluding pitch delivered per table.

The aim of the **planning phase** was to explore common ground and identify starting points for opportunities to improve health and wellbeing in Leiden Zuidwest. These opportunities had been identified in the previous phases and were grounded in the needs and aspirations of residents and professionals. Collected needs, aspirations, and opportunities were synthesized and visualized for greater accessibility to residents, professionals, and stakeholders, who were invited to reflect on the results and to reach agreement on activities to improve health and wellbeing in the neighbourhood.

During the whole study, the researchers established relationships with residents and professionals in the neighbourhood. They invested in being visible and accessible for residents and professionals. To facilitate impact at the meso-policy level, the researchers also engaged with policymakers and directors of organisations who are committed with the neighbourhood.

### Participants

A total of 150 adult residents and 50 professionals were involved in the study in the pre-orientation phase. A total of 50 residents and 30 professionals took part in the orientation and planning phases. The participating residents, aged between 40 and 90, represented a diverse mix of cultural backgrounds and lived throughout the neighbourhood. During the pre-orientation phase, relationships were gradually established with residents, creating trust and enabling more in-depth conversations over time. As this trust developed, residents increasingly contributed to articulating needs, aspirations, and opportunities, and some became actively involved in shaping and initiating follow-up activities. Their level of participation and empowerment thus evolved throughout the study, consistent with the iterative nature of participatory action research.

The professionals came from a diverse range of sectors and organizations. Primary healthcare providers included a neighbourhood collaboration with a coordinator, general practitioners, nurses, pharmacists, physiotherapists, dietitians and occupational therapists. Two home care organizations and two residential care centres also contributed. From the welfare sector, professionals from four different welfare organizations participated, including directors, social workers, and community sports coaches. Additionally, stakeholders from sectors indirectly influencing health and well-being – such as local entrepreneurs, police, housing corporations, and municipal policymakers – participated. While most professionals worked at the neighbourhood level, policymakers and directors operating at the meso-policy level participated in all phases of the study, ensuring a broader perspective on local health and welfare challenges.

### Data analysis

During observations and structured and unstructured interviews, researchers and students collected data through detailed field notes and photographs instead of audio and video recordings. This approach was chosen to preserve the informal and approachable nature of spontaneous encounters during the pre-orientation phase, when the aim was to build relationships and establish trust with a diverse group of residents and professionals. Introducing audio or video recording at this stage could have reduced openness and limited participants’ willingness to speak freely [36]. During transect walks among 25 residents, data were gathered using a combination of audio recordings, notes, and photographs. Data analysis was supported by ATLAS.ti, categorising identified needs, aspirations, and opportunities according to the domains for community prevention defined by the Dutch Ministry of Health, Welfare and Sport: (1) health and lifestyle, (2) facilities, (3) physical environment, (4) social environment, (5) participation, and (6) population [36]. The researchers introduced an additional domain, (7) working together; to capture emerging themes related to interprofessional cooperation between health and social professionals. Findings were presented during two Dream Laboratories and throughout the planning phase to elaborate on and enrich the data, as well as to prioritise preliminary results. Ultimately, the study outcomes were evaluated within the IPCHS framework, enabling a comparison between community-generated insights and the theoretical foundation.

The research proposal “Gezond en Inclusief Leiden Zuidwest” was reviewed and approved in April 2024 by the Ethical Research Committee of the Leiden University of Applied Sciences (File no. 20240405 Guijt). The committee concluded that the study complies with the criteria of the Dutch Code of Conduct for Research Integrity. All participants were informed about the purpose of the study, and participation was voluntary. For research activities involving audio recording, such as the transect walks and Dream Laboratories, written informed consent was obtained prior to participation.

## Results

### Identified needs, aspirations, and opportunities

At the end of the planning phase, the identified needs, aspirations, and opportunities were made publicly available in the Atlas of the Neighbourhood (*Wijkatlas* in Dutch) and the Treasure Map (*Schatkaart* in Dutch) of Leiden Zuidwest. The ‘Atlas’ presents an overview of residents’ and professionals’ input on local needs and aspirations, enriched with demographic data, social characteristics, and relevant policy information. A summary of needs, aspirations, and opportunities were compiled into the ‘Treasure Map’. Together, participants identified three central themes to guide future collaborative efforts to improve health and wellbeing in the neighbourhood:

1. **Interdisciplinary Collaboration:** How can health and social care professionals work together more effectively?
2. **Citizen Participation:** How can residents participate more actively and meaningfully in neighbourhood life?
3. **Awareness of Available Services:** How can the existing services in Leiden Zuidwest be better communicated and utilized?

These themes serve as a foundation to collaboratively develop targeted actions.

### Valuable unintended effects

In addition to identifying needs, aspirations, and opportunities, three valuable unintended effects began to emerge during the process: the development of relationships between the research team and participants, enhanced collaboration among health and wellbeing professionals, and initial improvements in neighbourhood services. First, relationships formed between researchers, residents, and professionals. During the pre-orientation phase, conversations remained surface-level, with participants sharing only superficial needs and aspirations. However, as trust developed, they began to express deeper, more personal needs. Initially, researchers initiated all interactions, but over time, residents and professionals began inviting them to meetings, activities and discussions within the neighbourhood. For example, a physiotherapist and residents of a seniors’ apartment building asked a researcher to assist them in setting up an exercise group. Furthermore, the primary care collaboration funded the second Dream Laboratory. These developments signalled increasing commitment from the community and a growing recognition of the value of action research and researcher involvement. Additionally, the researchers engaged with directors and policymakers at the meso-policy level, thus strengthening involvement not only locally but also at a higher decision-making level. Researchers acted as a bridge between residents and professionals while also linking residents and professionals with directors and policymakers on the meso-policy level. A tangible example of this is the Atlas, which was later used by the municipality and housing corporation to inform new plans and policies.

Secondly, relationships between participating health and wellbeing service professionals were strengthened during the research. Prior to the study, most of them did not know each other. Several dedicated meetings accelerated contact and collaboration between them. For example, the primary care collaboration manager came into contact with social workers from all welfare organisations during the Dream Laboratories. Now they invite each other to relevant meetings, share updates and information about activities in the neighbourhood, coordinate meetings, align website content, and share the use of an app informing about all exercise and meeting facilities.

Lastly, this study brought initial improvements in health and wellbeing services in the neighbourhood. For example, residents who participated in a transect walk and visited the Dream Laboratory, started an accessible and for-free exercise group for residents – ‘Vitality Club’ – led by a peer coach who is not a professional. Residents themselves have set up the group and provide exercises. The researchers encouraged residents and offered support where necessary.

### IPCHS framework

This PAR started with the IPCHS strategy ‘engaging and empowering people and the community’. Residents and various professionals were not only consulted, but also actively engaged in all phases. This engagement led to the establishment of long-term relationships with researchers and other participants, thereby providing opportunities and resources for transforming the participants’ own neighbourhood.

Opportunities and improvements also emerged within the IPCHS strategy: ‘coordinating services within and across sectors’, aimed at better alignment of health and wellbeing services. The overview of needs, aspirations, and opportunities in the Atlas and Treasure Map helped coordinate suitable services. Furthermore, the study contributed to strengthened and accelerated intersectoral connections and collaborations.

Lastly, this study represented a starting point for the IPCHS strategy: ‘creating an enabling environment and financial support’. While stakeholders collectively agreed on three main themes to improve health and wellbeing in the neighbourhood, the primary health neighbourhood collaboration and welfare organizations, for example, were willing to help fund meetings.

No improvements were seen yet in the IPCHS strategies ‘Strengthening governance and accountability’ and ‘Reorienting the model of care’.

## Discussion

This case study presents a novel, community-up approach to uncover and understand the needs and aspirations of both residents and professionals, while identifying opportunities to enhance health and wellbeing in a Dutch neighbourhood. This process led to the development of two visual products: an *Atlas of the Neighbourhood* and a *Treasure Map*, which highlighted potential avenues for planning and development initiatives. Additionally, the approach yielded valuable, unintended outcomes, such as development of relationships, enhanced collaboration, and initial improvements in neighbourhood services. These results aligned with the following IPCHS strategies: 1. engaging and empowering people and communities, 2. coordinating services within and across sectors, and 3. creating an enabling environment and financial support.

What sets this approach apart is the distinctive role of action researchers from the UASL, the use of a diverse range of generative methods and creative products (the Atlas and Treasure Map), and the support of students in data collection. Ravanghi et al. [2023] noted common challenges in mapping health needs, such as professionals lacking the time or skills to conduct neighbourhood-based assessments as well as the potential for their own professional interests to influence outcomes [20,37].

In contrast, our research team – comprising professionals with backgrounds in health, social care, and PAR – dedicated a full year to identifying local needs and aspirations. Acting as independent yet critically engaged allies, the researchers focused on listening without judgment, which helped build trust with both residents and professionals. The use of generative and creative PAR methods, exemplified by the Atlas and the Treasure Map, made opportunities for positive change more tangible and accessible, enhancing participant engagement, ownership, and dialogue around health and wellbeing in a more inclusive way. The involvement of students encouraged participation and attracted a diverse group of residents; for example, individuals from various cultural backgrounds and age groups noted that the students’ enthusiasm motivated them to join a transect walk and participate in the Dream Laboratory meeting.

A distinguishing feature of our study is the emergence of valuable additional effects, particularly the active role played by researchers in engaging residents, local professionals, and stakeholders at the meso-policy level, including directors and policymakers. Although this was not a predefined objective, it proved to be very important. Engagement can be characterized by who is interacting with whom, and at what level [38]. Boivin et al. [2022] distinguishes three types of engagement: bonding, bridging and linking. These forms of engagement can be understood within the broader theoretical framework of social capital, which differentiates between 1. Bonding refers to ‘inward looking’ connections among residents or professionals, while 2. bridging refers to ‘looking across’ connections between residents and professional groups. Furthermore, 3. linking refers to ‘upward looking’ connections across power authority and levels [38,39]. In this PAR project, UASL researchers functioned as a vital linking pin across all three forms of engagement. During the pre-orientation phase, introductory conversations and meetings resulted in unexpected bonding between researchers and both residents and professionals. In the subsequent phases, ongoing meetings facilitated bridging connections – both between residents and professionals and among professionals from different sectors. Simultaneously, researchers cultivated bonding connections with policymakers and directors at the meso-policy level, which provided an unexpected linking role between local residents and engaged directors and policymakers.

The IPCHS framework served as the theoretical lens to evaluate the outcomes of the PAR project, and the findings aligned with three of the five IPCHS strategies. The framework was applied retrospectively, which may explain the absence of findings aligned with the remaining strategies, dealing with governance. Another contributing factor could be the health and welfare scope of the research team. Within this PAR, active involvement and alignment were achieved with the municipality, but steps towards governance and accountability have not yet been taken. Nevertheless, this case study provides opportunities to strengthen governance and reorienting the model of care in Leiden Zuidwest. Moreover, it underscores the importance of building on the outcomes related to the other three strategies of the IPCHS framework. This study represents an initial step towards integrated people-centred health care services, although historical lessons remind us that establishing high-quality, people-centred integrated care is likely to be a long journey – one that requires sustained political commitment [1,17].

Some limitations should be noted. Although Leiden Zuidwest has a diverse population of over 20,000, 150 residents participated in this study – representing various age groups, cultural backgrounds, and areas within the neighbourhood. While PAR promotes inclusivity, the relatively small sample may not fully capture the neighbourhood’s range of needs and aspirations. As such, the Atlas and Treasure Map should be seen not as an absolute representation of reality, but as a starting point for ongoing dialogue between residents and professionals around health and wellbeing. Moreover, in the Atlas and the Treasure Map, complex and fragmented information drawn from policy documents, demographic reports, and professional records was brought together and presented in a structured and accessible way. Through the use of visuals, maps, and clear language, these visuals supports shared understanding and facilitates more inclusive and accessible dialogue about the neighbourhood [40]. They are dynamic tools, intended to evolve with new insights. In addition, although the IPCHS framework was applied retrospectively as a theoretical lens, its use may have influenced the interpretation and organisation of the findings. Despite efforts to remain open to emergent themes, the framework may have directed attention toward domains aligned with integrated care, potentially shaping the way the data were structured and interpreted. A further challenge was the limited availability of professionals. Busy schedules, differing compensation structures, and varied availability often made it difficult to bring professionals together simultaneously. Nevertheless, all health and social care professionals in the neighbourhood were engaged, and there is increasing focus on strengthening connections and collaboration.

This study demonstrated a novel community-up approach to identify the needs and aspirations of residents and professionals, creating opportunities aligned with IPCHS strategies in a high-income country urban neighbourhood. Beyond insights, the approach also fostered greater engagement and use of health and wellbeing services, suggesting its potential as an intervention. In addition to the intended outcomes, several unintended impacts emerged, including bonding, bridging and linking, trust-building and strengthened collaboration among stakeholders. In line with the Position Paper on Impact in Participatory Health Research, such unintended impacts are meaningful changes and include shifts in thinking, actions, and relationships among researchers, practitioners, and community members [41].

Further research is required to explore its applicability in other neighbourhoods, considering the role of action researchers and multi-level engagement. Additionally, follow-up research is needed to assess the longer term impact. Capturing both intended and unintended impacts remains complex, particularly as universally accepted indicators for capturing the impact of IPCHS strategies are still lacking [1].

## Lessons learned

- **Value of PAR methods:** Participatory action research, combined with generative and creative methods, yields rich insights into the needs, aspirations, and opportunities for improving health and wellbeing in neighbourhoods. The use of accessible visuals facilitated more inclusive and accessible dialogue, increasing engagement, participation, and a sense of ownership among participants.
- **Importance of long-term engagement:** Addressing health and wellbeing at the neighbourhood level requires sustained relationships and early, continuous involvement of both residents and professionals. Building these relationships is complex and time-intensive because it requires balancing differing interests, availability and financial expectations. Leveraging existing meetings and networks can support this process.
- **Role of Applied University researchers:** Researchers from Universities of Applied Sciences can play a key role – not only by contributing research expertise but also by acting as independent allies who bond, bridge, and link residents and professionals across sectors. Universities of Applied Sciences are strongly embedded in local professional networks and are characterised by a practice-oriented orientation, enabling them to remain closely connected to professional practice and contemporary challenges in health and social care. Their involvement, along with that of students, can help engage a diverse group of participants and generate rich, wide-ranging insights.

## Conclusion

This study demonstrated a novel community-up approach to identifying needs, aspirations, and opportunities to improve health and wellbeing at the neighbourhood level, contributing meaningfully to three IPCHS strategies: 1. engaging and empowering people and communities, 2. coordinating services within and across sectors, and 3. creating an enabling environment and financial support. Additionally, the involvement of researchers from a University of Applied Sciences proved to be an added value, acting as independent allies who bond, bridge, and link residents, professionals, and policymakers. Further research is needed to explore the applicability of this community-up approach in other neighbourhoods and to develop methods for capturing and demonstrating both intended and unintended impacts including relational and system-level changes that emerge through participatory processes.
